# Ethyl 2-(3,3-dibutyl­thio­ureido)-4,5,6,7-tetra­hydro­benzo[*b*]thio­phene-3-carboxyl­ate

**DOI:** 10.1107/S1600536809047230

**Published:** 2009-11-14

**Authors:** Hong-Mei Wang, Jing Xu, Xiao-Hua Zeng, Jia-Hua Tian

**Affiliations:** a Institute of Medicinal Chemistry, Yunyang Medical College, Shiyan 442000, People’s Republic of China

## Abstract

In the title compound, C_20_H_32_N_2_O_2_S_2_, the cyclo­hexene ring is disordered over two half-boat conformations with occupancy factors of 0.71:0.29. One *n*-butyl chain is also disordered over two positions with occupancy factors of 0.83:0.17. The mol­ecular conformation is stabilized by an intra­molecular N—H⋯O hydrogen bond.

## Related literature

For the synthesis and biological activity of thienopyrimidin-4(3*H*)-one derivatives, see: De Laszlo *et al.* (1992*a* ▶,*b* ▶); Taguchi *et al.* (1993*a* ▶,*b* ▶); Walter (1999*a* ▶,*b* ▶); Ding *et al.* (2004 ▶); Santagati *et al.* (2003 ▶); Abbott GmbH Co KG (2004*a* ▶, 2004*b* ▶); Walter & Zeun (2004 ▶); Ford *et al.* (2004*a* ▶,*b* ▶); Duval *et al.* (2005 ▶); Waehaelae *et al.* (2004*a* ▶,*b* ▶). For a description of the Cambridge Structural Database, see: Allen (2002 ▶). For related structures, see: Xu *et al.* (2005 ▶); Zeng *et al.* (2005 ▶, 2006 ▶, 2007 ▶, 2008 ▶, 2009 ▶); Wang *et al.* (2007 ▶, 2008 ▶); Zheng *et al.* (2007 ▶); Xie *et al.* (2008 ▶).
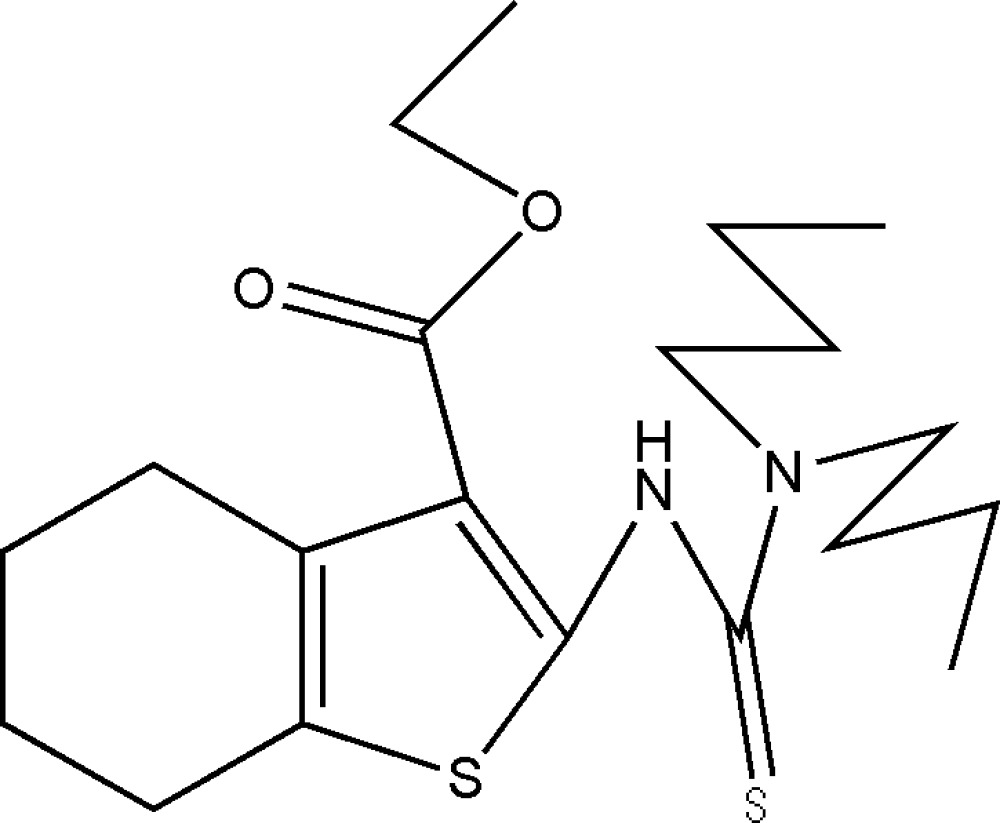


## Experimental

### 

#### Crystal data

C_20_H_32_N_2_O_2_S_2_
*M* *_r_* = 396.60
Monoclinic, 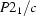

*a* = 10.9311 (11) Å
*b* = 21.715 (3) Å
*c* = 9.6841 (3) Å
β = 107.711 (12)°
*V* = 2189.8 (4) Å^3^
*Z* = 4
Mo *K*α radiation
μ = 0.26 mm^−1^
*T* = 292 K
0.36 × 0.30 × 0.25 mm

#### Data collection

Bruker SMART CCD area-detector diffractometer
Absorption correction: multi-scan (*SADABS*; Sheldrick, 1996 ▶) *T*_min_ = 0.903, *T*_max_ = 0.938
12194 measured reflections
4036 independent reflections
3460 reflections with *I* > 2σ(*I*)
*R*_int_ = 0.027

#### Refinement

*R*[*F* ^2^ > 2σ(*F* ^2^)] = 0.073
*wR*(*F* ^2^) = 0.153
*S* = 1.21
4036 reflections
280 parameters
13 restraints
H atoms treated by a mixture of independent and constrained refinement
Δρ_max_ = 0.30 e Å^−3^
Δρ_min_ = −0.23 e Å^−3^

### 

Data collection: *SMART* (Bruker, 2001 ▶); cell refinement: *SAINT* (Bruker, 2001 ▶); data reduction: *SAINT*; program(s) used to solve structure: *SHELXS97* (Sheldrick, 2008 ▶); program(s) used to refine structure: *SHELXL97* (Sheldrick, 2008 ▶); molecular graphics: *PLATON* (Spek, 2009 ▶); software used to prepare material for publication: *SHELXTL97* (Sheldrick, 2008 ▶).

## Supplementary Material

Crystal structure: contains datablocks global, I. DOI: 10.1107/S1600536809047230/rz2377sup1.cif

Structure factors: contains datablocks I. DOI: 10.1107/S1600536809047230/rz2377Isup2.hkl

Additional supplementary materials:  crystallographic information; 3D view; checkCIF report

## Figures and Tables

**Table 1:** Hydrogen-bond geometry (Å, °)

*D*—H⋯*A*	*D*—H	H⋯*A*	*D*⋯*A*	*D*—H⋯*A*
N1—H1*A*⋯O2	0.86 (3)	1.89 (2)	2.643 (4)	145 (3)

**Fig. 1.**
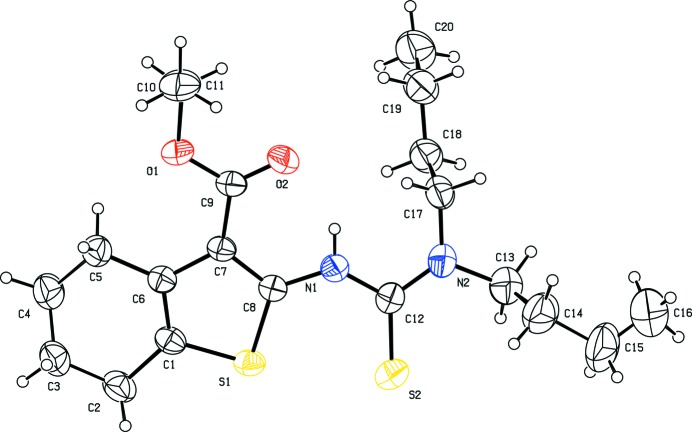
The molecular structure of the title compound showing the atom-labeling scheme. Displacement ellipsoids are drawn at 50% probability level. H atoms are represented by circles of arbitrary size. Only the major components of the disordered groups are shown.

## supplementary crystallographic information

### Comment

The derivatives of heterocycles containing the thienopyrimidine system, which are well known bioisosteres of quinazolines, are of great importance because of their remarkable biological properties, including antimicrobial or antifungal activities (De Laszlo *et al.*, 1992*a*,*b*; Walter, 1999*a*,*b*; Ding *et al.*, 2004; Walter & Zeun, 2004), significant 5-HT_1A_ and 5-HT_1B_ receptor activities (Taguchi *et al.*, 1993*a*,*b*; Abbott GmbH & Co. KG, 2004*a*,*b*), potential selective COX-2 enzyme inhibitor activity (Santagati, *et al.*, 2004), 17beta-hydroxysteroid dehydrogenase inhibitor activity (Waehaelae *et al.*, 2004*a*,*b*), potassium channel inhibitor activity (Ford *et al.*, 2004*a*,*b*), and tissue transglutaminase inhibitor activity (Duval *et al.*, 2005). Recently, our group has been engaged in the preparation of derivatives of thienopyrimidin-4(3*H*)-one *via* aza-Wittig reaction of beta-ethoxycarbonyl iminophosphorane with CS_2_. As a continuation of our research for new biologically active heterocycles, the title compound was obtained as an intermediate product from beta-ethoxycarbonyl iminophosphorane in CS_2_ and structurally characterized in order to elucidate the cyclization mechanism involved in the reaction.

In the title compound (Fig. 1), bond lengths within the benzothiophene ring system are in good agreement with those observed for closely related structures (Xu *et al.*, 2005; Zeng *et al.*, 2005, 2006, 2007, 2008, 2009; Wang *et al.*, 2007, 2008; Zheng *et al.*, 2007; Xie *et al.*, 2008), and in the ranges of values observed in previously reported structures in the Cambridge Structural Database (Version 5.26; Allen, 2002). The theiophene ring is planar, with a maximum displacement of 0.008 (3)Å for atom C8. The attached cyclohexene ring is disordered over two half-chair conformations with site occupancy factors of 0.71:0.29. A *n*-butyl chain is also disordered over two positions with site occupancy factors of 0.83:0.17. The molecular conformation is stabilized by an intramolecular N—H···O hydrogen bond (Table 1). The crystal packing is enforced only by van der Waals interactions.

### Experimental

To a solution of ethyl 2-thiocyanato-4,5,6,7-tetrahydrobenzo[*b*]thiophene -3-carboxylate (3 mmol) prepared according to Zeng *et al.* (2005) in CH_3_CN (15 ml) was added dibutylamine (3 mmol) at room temperature, and the reaction mixture was stirred for 6 h. The solvent was removed under reduced pressure and the residue was recrystallized from EtOH to give the title compound in yield of 81% (m.p. 442 K). Elemental analysis:calculated for C_20_H_32_N_2_O_2_S_2_: C, 60.57; H, 8.13; N, 7.06. Found: C, 59.34; H, 8.55; N, 6.67%. Crystals suitable for X-ray diffraction analysis were obtained by slow evaporation of a hexane/dichloromethane (1:3 *v*/*v*) solution at room temperature.

### Refinement

The C4, C5 carbon atoms of the cyclohexene ring and the C15, C16 carbon atoms of one *n*-butyl chain are disordered over two positions with site occupancy factors of 0.71:0.29 and 0.83:0.17, respectively. During the refinement, the C—C bond lengths involving the disordered carbon atoms have been constrained to be 1.54 (1) Å. The H atom attached to atom N1 was located in a difference Fourier map and allowed to ride with a restraint of N—H = 0.86 Å. Other H atoms were placed at calculated positions and treated as riding atoms, with C—H = 0.96–0.97 Å, and *U*_iso_(H) = 1.2 *U*_eq_(C) or 1.5 *U*_eq_(C) for methyl H atoms.

### Crystal data

**Table:**

C_20_H_32_N_2_O_2_S_2_	*F*(000) = 856
*M**_r_* = 396.60	*D*_x_ = 1.203 Mg m^−^^3^
Monoclinic, *P*2_1_/*c*	Mo *K*α radiation, λ = 0.71073 Å
Hall symbol: -P 2ybc	Cell parameters from 9055 reflections
*a* = 10.9311 (11) Å	θ = 2.2–27.6°
*b* = 21.715 (3) Å	µ = 0.26 mm^−^^1^
*c* = 9.6841 (3) Å	*T* = 292 K
β = 107.711 (12)°	Block, colourless
*V* = 2189.8 (4) Å^3^	0.36 × 0.30 × 0.25 mm
*Z* = 4	

### Data collection

**Table:**

Bruker SMART CCD area-detector diffractometer	4036 independent reflections
Radiation source: fine-focus sealed tube	3460 reflections with *I* > 2σ(*I*)
graphite	*R*_int_ = 0.027
φ and ω scans	θ_max_ = 25.5°, θ_min_ = 2.1°
Absorption correction: multi-scan (*SADABS*; Sheldrick, 1996)	*h* = −13→13
*T*_min_ = 0.903, *T*_max_ = 0.938	*k* = −21→26
12194 measured reflections	*l* = −11→11

### Refinement

**Table:**

Refinement on *F*^2^	Primary atom site location: structure-invariant direct methods
Least-squares matrix: full	Secondary atom site location: difference Fourier map
*R*[*F*^2^ > 2σ(*F*^2^)] = 0.073	Hydrogen site location: inferred from neighbouring sites
*wR*(*F*^2^) = 0.153	H atoms treated by a mixture of independent and constrained refinement
*S* = 1.21	*w* = 1/[σ^2^(*F*_o_^2^) + (0.0275*P*)^2^ + 2.0031*P*] where *P* = (*F*_o_^2^ + 2*F*_c_^2^)/3
4036 reflections	(Δ/σ)_max_ < 0.001
280 parameters	Δρ_max_ = 0.30 e Å^−^^3^
13 restraints	Δρ_min_ = −0.23 e Å^−^^3^

### Special details

**Table:**

Geometry. All e.s.d.'s (except the e.s.d. in the dihedral angle between two l.s. planes) are estimated using the full covariance matrix. The cell e.s.d.'s are taken into account individually in the estimation of e.s.d.'s in distances, angles and torsion angles; correlations between e.s.d.'s in cell parameters are only used when they are defined by crystal symmetry. An approximate (isotropic) treatment of cell e.s.d.'s is used for estimating e.s.d.'s involving l.s. planes.
Refinement. Refinement of *F*^2^ against ALL reflections. The weighted *R*-factor *wR* and goodness of fit *S* are based on *F*^2^, conventional *R*-factors *R* are based on *F*, with *F* set to zero for negative *F*^2^. The threshold expression of *F*^2^ > σ(*F*^2^) is used only for calculating *R*-factors(gt) *etc*. and is not relevant to the choice of reflections for refinement. *R*-factors based on *F*^2^ are statistically about twice as large as those based on *F*, and *R*- factors based on ALL data will be even larger.

### Fractional atomic coordinates and isotropic or equivalent isotropic displacement parameters (Å^2^)

**Table:**

	*x*	*y*	*z*	*U*_iso_*/*U*_eq_	Occ. (<1)
C1	1.0984 (4)	−0.00230 (16)	0.7833 (4)	0.0631 (9)	
C2	1.1175 (5)	−0.0488 (2)	0.9023 (5)	0.0856 (12)	
H2A	1.0446	−0.0765	0.8804	0.103*	0.71
H2B	1.1237	−0.0280	0.9928	0.103*	0.71
H2C	1.0714	−0.0361	0.9687	0.103*	0.29
H2D	1.0832	−0.0882	0.8616	0.103*	0.29
C3	1.2396 (7)	−0.0852 (4)	0.9174 (11)	0.097 (2)	0.71
H3A	1.2660	−0.1048	1.0119	0.117*	0.71
H3B	1.2207	−0.1175	0.8448	0.117*	0.71
C4	1.3461 (8)	−0.0488 (4)	0.9018 (8)	0.093 (2)	0.71
H4A	1.4202	−0.0754	0.9147	0.112*	0.71
H4B	1.3686	−0.0177	0.9771	0.112*	0.71
C4'	1.315 (2)	−0.0763 (7)	0.8441 (17)	0.077 (5)	0.29
H4'1	1.4018	−0.0918	0.8832	0.092*	0.29
H4'2	1.2627	−0.1081	0.7835	0.092*	0.29
C3'	1.2569 (14)	−0.0586 (11)	0.9758 (17)	0.113 (9)	0.29
H3'1	1.2705	−0.0918	1.0458	0.135*	0.29
H3'2	1.2968	−0.0215	1.0251	0.135*	0.29
C5	1.3142 (4)	−0.01737 (18)	0.7532 (4)	0.0751 (10)	
H5A	1.3787	0.0136	0.7554	0.090*	0.71
H5B	1.3163	−0.0476	0.6803	0.090*	0.71
H5C	1.3789	0.0116	0.8067	0.090*	0.29
H5D	1.3339	−0.0286	0.6653	0.090*	0.29
C6	1.1838 (3)	0.01239 (15)	0.7127 (3)	0.0577 (8)	
C7	1.1347 (3)	0.05951 (15)	0.6064 (3)	0.0551 (8)	
C8	1.0133 (3)	0.07897 (14)	0.6033 (3)	0.0542 (7)	
C9	1.1975 (3)	0.08581 (17)	0.5067 (4)	0.0634 (9)	
C10	1.3820 (4)	0.0835 (2)	0.4231 (5)	0.0951 (14)	
H10A	1.3581	0.1259	0.3967	0.114*	
H10B	1.4735	0.0824	0.4730	0.114*	
C11	1.3540 (5)	0.0462 (2)	0.2931 (5)	0.1069 (16)	
H11A	1.3741	0.0040	0.3193	0.160*	
H11B	1.4048	0.0602	0.2340	0.160*	
H11C	1.2646	0.0498	0.2398	0.160*	
C12	0.8369 (3)	0.15459 (17)	0.5066 (4)	0.0659 (9)	
C13	0.6951 (5)	0.2433 (2)	0.4072 (6)	0.1029 (15)	
H13A	0.6756	0.2414	0.4983	0.123*	
H13B	0.7147	0.2858	0.3911	0.123*	
C14	0.5827 (5)	0.2238 (3)	0.2904 (6)	0.1242 (19)	
H14A	0.5686	0.1800	0.2980	0.149*	0.83
H14B	0.5957	0.2317	0.1973	0.149*	0.83
H14C	0.5373	0.1905	0.3203	0.149*	0.17
H14D	0.6066	0.2106	0.2064	0.149*	0.17
C15	0.4615 (9)	0.2616 (5)	0.3037 (12)	0.181 (5)	0.83
H15A	0.3847	0.2396	0.2496	0.217*	0.83
H15B	0.4641	0.2612	0.4047	0.217*	0.83
C16	0.4496 (12)	0.3174 (5)	0.2607 (16)	0.232 (7)	0.83
H16A	0.5305	0.3381	0.2977	0.348*	0.83
H16B	0.3864	0.3376	0.2953	0.348*	0.83
H16C	0.4228	0.3184	0.1567	0.348*	0.83
C15'	0.4923 (18)	0.2749 (12)	0.213 (3)	0.090 (8)	0.17
H15C	0.5365	0.3140	0.2372	0.108*	0.17
H15D	0.4727	0.2690	0.1094	0.108*	0.17
C16'	0.372 (2)	0.2782 (14)	0.248 (5)	0.125 (13)	0.17
H16D	0.3180	0.3090	0.1892	0.188*	0.17
H16E	0.3897	0.2888	0.3487	0.188*	0.17
H16F	0.3298	0.2390	0.2303	0.188*	0.17
C17	0.9008 (4)	0.23002 (19)	0.3483 (5)	0.0814 (11)	
H17A	0.8536	0.2535	0.2637	0.098*	
H17B	0.9449	0.1968	0.3162	0.098*	
C18	0.9998 (4)	0.2719 (2)	0.4523 (5)	0.0897 (13)	
H18A	0.9553	0.3058	0.4813	0.108*	
H18B	1.0437	0.2487	0.5388	0.108*	
C19	1.0961 (5)	0.2970 (2)	0.3882 (5)	0.1017 (15)	
H19A	1.0523	0.3158	0.2955	0.122*	
H19B	1.1485	0.2635	0.3709	0.122*	
C20	1.1828 (5)	0.3447 (2)	0.4862 (6)	0.126 (2)	
H20A	1.1321	0.3792	0.4981	0.190*	
H20B	1.2465	0.3582	0.4432	0.190*	
H20C	1.2244	0.3266	0.5791	0.190*	
N1	0.9493 (3)	0.12572 (14)	0.5138 (3)	0.0637 (7)	
H1A	0.997 (3)	0.1387 (16)	0.464 (3)	0.076*	
N2	0.8108 (3)	0.20414 (16)	0.4177 (4)	0.0801 (9)	
O1	1.3122 (2)	0.06116 (13)	0.5196 (3)	0.0769 (7)	
O2	1.1519 (3)	0.12628 (13)	0.4189 (3)	0.0869 (8)	
S1	0.95706 (9)	0.03954 (4)	0.72506 (10)	0.0662 (3)	
S2	0.74212 (10)	0.13005 (5)	0.60210 (13)	0.0823 (3)	

### Atomic displacement parameters (Å^2^)

**Table:**

	*U*^11^	*U*^22^	*U*^33^	*U*^12^	*U*^13^	*U*^23^
C1	0.080 (2)	0.060 (2)	0.0549 (19)	−0.0045 (17)	0.0284 (17)	−0.0010 (16)
C2	0.106 (3)	0.085 (3)	0.075 (3)	0.006 (2)	0.041 (2)	0.018 (2)
C3	0.109 (6)	0.097 (6)	0.093 (6)	0.026 (4)	0.040 (5)	0.035 (5)
C4	0.094 (5)	0.099 (6)	0.083 (6)	0.014 (5)	0.021 (5)	0.019 (4)
C4'	0.083 (12)	0.078 (11)	0.068 (10)	0.020 (9)	0.021 (9)	−0.004 (8)
C3'	0.18 (2)	0.106 (17)	0.053 (10)	0.055 (16)	0.040 (12)	0.009 (9)
C5	0.079 (2)	0.074 (2)	0.077 (3)	0.010 (2)	0.030 (2)	0.006 (2)
C6	0.0649 (19)	0.0570 (19)	0.0541 (18)	−0.0018 (16)	0.0225 (15)	−0.0035 (15)
C7	0.0634 (19)	0.0523 (17)	0.0542 (18)	−0.0054 (15)	0.0248 (15)	−0.0023 (14)
C8	0.0610 (18)	0.0520 (18)	0.0511 (17)	−0.0049 (15)	0.0194 (15)	−0.0051 (14)
C9	0.068 (2)	0.062 (2)	0.067 (2)	−0.0079 (17)	0.0303 (18)	0.0015 (18)
C10	0.081 (3)	0.113 (4)	0.108 (4)	−0.012 (3)	0.053 (3)	0.013 (3)
C11	0.111 (4)	0.130 (4)	0.102 (4)	−0.005 (3)	0.065 (3)	0.004 (3)
C12	0.062 (2)	0.065 (2)	0.068 (2)	0.0013 (17)	0.0149 (17)	−0.0078 (18)
C13	0.098 (3)	0.094 (3)	0.116 (4)	0.013 (3)	0.031 (3)	0.008 (3)
C14	0.090 (4)	0.154 (5)	0.118 (4)	0.010 (4)	0.015 (3)	0.014 (4)
C15	0.111 (7)	0.256 (15)	0.161 (9)	0.068 (8)	0.021 (6)	0.083 (9)
C16	0.180 (11)	0.235 (14)	0.296 (17)	0.081 (11)	0.096 (11)	0.137 (14)
C15'	0.09 (2)	0.087 (19)	0.09 (2)	0.015 (16)	0.029 (17)	0.017 (16)
C16'	0.10 (3)	0.10 (2)	0.18 (4)	−0.02 (2)	0.05 (3)	0.03 (2)
C17	0.084 (3)	0.076 (3)	0.079 (3)	0.008 (2)	0.017 (2)	0.016 (2)
C18	0.092 (3)	0.086 (3)	0.087 (3)	−0.001 (2)	0.020 (2)	0.009 (2)
C19	0.109 (4)	0.089 (3)	0.104 (4)	−0.005 (3)	0.027 (3)	0.015 (3)
C20	0.119 (4)	0.102 (4)	0.137 (5)	−0.025 (3)	0.007 (4)	0.003 (4)
N1	0.0641 (17)	0.0633 (18)	0.0680 (19)	0.0034 (14)	0.0262 (14)	0.0037 (15)
N2	0.073 (2)	0.076 (2)	0.090 (2)	0.0137 (17)	0.0216 (18)	0.0066 (19)
O1	0.0683 (15)	0.0907 (19)	0.0836 (18)	−0.0001 (14)	0.0410 (14)	0.0126 (14)
O2	0.094 (2)	0.0862 (19)	0.095 (2)	0.0115 (16)	0.0501 (17)	0.0313 (16)
S1	0.0714 (6)	0.0700 (6)	0.0664 (6)	−0.0047 (4)	0.0345 (4)	0.0008 (4)
S2	0.0682 (6)	0.0924 (8)	0.0923 (8)	−0.0011 (5)	0.0333 (5)	−0.0054 (6)

### Geometric parameters (Å, °)

**Table:**

C1—C6	1.352 (4)	C12—N2	1.353 (5)
C1—C2	1.498 (5)	C12—N1	1.362 (4)
C1—S1	1.732 (4)	C12—S2	1.672 (4)
C2—C3'	1.488 (15)	C13—C14	1.457 (6)
C2—C3	1.520 (8)	C13—N2	1.502 (5)
C2—H2A	0.9700	C13—H13A	0.9700
C2—H2B	0.9700	C13—H13B	0.9700
C2—H2C	0.9700	C14—C15'	1.522 (16)
C2—H2D	0.9700	C14—C15	1.597 (9)
C3—C4	1.452 (11)	C14—H14A	0.9700
C3—H3A	0.9700	C14—H14B	0.9700
C3—H3B	0.9700	C14—H14C	0.9700
C4—C5	1.534 (7)	C14—H14D	0.9700
C4—H4A	0.9700	C15—C16	1.274 (11)
C4—H4B	0.9700	C15—H15A	0.9700
C4'—C5	1.552 (14)	C15—H15B	0.9700
C4'—C3'	1.63 (2)	C16—H16A	0.9600
C4'—H4'1	0.9700	C16—H16B	0.9600
C4'—H4'2	0.9700	C16—H16C	0.9600
C3'—H3'1	0.9700	C15'—C16'	1.454 (19)
C3'—H3'2	0.9700	C15'—H15C	0.9700
C5—C6	1.505 (5)	C15'—H15D	0.9700
C5—H5A	0.9700	C16'—H16D	0.9600
C5—H5B	0.9700	C16'—H16E	0.9600
C5—H5C	0.9700	C16'—H16F	0.9600
C5—H5D	0.9700	C17—N2	1.461 (5)
C6—C7	1.435 (4)	C17—C18	1.532 (6)
C7—C8	1.385 (4)	C17—H17A	0.9700
C7—C9	1.460 (4)	C17—H17B	0.9700
C8—N1	1.380 (4)	C18—C19	1.480 (6)
C8—S1	1.715 (3)	C18—H18A	0.9700
C9—O2	1.219 (4)	C18—H18B	0.9700
C9—O1	1.333 (4)	C19—C20	1.524 (6)
C10—C11	1.449 (6)	C19—H19A	0.9700
C10—O1	1.458 (4)	C19—H19B	0.9700
C10—H10A	0.9700	C20—H20A	0.9600
C10—H10B	0.9700	C20—H20B	0.9600
C11—H11A	0.9600	C20—H20C	0.9600
C11—H11B	0.9600	N1—H1A	0.86 (3)
C11—H11C	0.9600		
			
C6—C1—C2	126.3 (4)	C10—C11—H11B	109.5
C6—C1—S1	113.3 (3)	H11A—C11—H11B	109.5
C2—C1—S1	120.4 (3)	C10—C11—H11C	109.5
C3'—C2—C1	110.3 (8)	H11A—C11—H11C	109.5
C1—C2—C3	109.7 (4)	H11B—C11—H11C	109.5
C3'—C2—H2A	132.0	N2—C12—N1	114.3 (3)
C1—C2—H2A	109.7	N2—C12—S2	124.0 (3)
C3—C2—H2A	109.7	N1—C12—S2	121.7 (3)
C3'—C2—H2B	81.6	C14—C13—N2	112.5 (4)
C1—C2—H2B	109.7	C14—C13—H13A	109.1
C3—C2—H2B	109.7	N2—C13—H13A	109.1
H2A—C2—H2B	108.2	C14—C13—H13B	109.1
C3'—C2—H2C	112.7	N2—C13—H13B	109.1
C1—C2—H2C	109.8	H13A—C13—H13B	107.8
C3—C2—H2C	134.6	C13—C14—C15'	116.0 (11)
H2A—C2—H2C	76.3	C13—C14—C15	108.2 (6)
C3'—C2—H2D	106.3	C13—C14—H14A	110.1
C1—C2—H2D	109.6	C15'—C14—H14A	131.1
C3—C2—H2D	78.4	C15—C14—H14A	110.1
H2B—C2—H2D	133.7	C13—C14—H14B	110.1
H2C—C2—H2D	107.8	C15—C14—H14B	110.1
C4—C3—C2	114.6 (6)	H14A—C14—H14B	108.4
C4—C3—H3A	108.6	C13—C14—H14C	111.9
C2—C3—H3A	108.6	C15'—C14—H14C	112.0
C4—C3—H3B	108.6	H14B—C14—H14C	130.0
C2—C3—H3B	108.6	C13—C14—H14D	111.0
H3A—C3—H3B	107.6	C15'—C14—H14D	95.9
C3—C4—C5	112.3 (7)	C15—C14—H14D	131.3
C3—C4—H4A	109.2	H14C—C14—H14D	108.9
C5—C4—H4A	109.2	C16—C15—C14	117.9 (11)
C3—C4—H4B	109.2	C16—C15—H15A	107.8
C5—C4—H4B	109.2	C14—C15—H15A	107.8
H4A—C4—H4B	107.9	C16—C15—H15B	107.8
C5—C4'—C3'	108.2 (12)	C14—C15—H15B	107.8
C5—C4'—H4'1	110.1	H15A—C15—H15B	107.2
C3'—C4'—H4'1	110.1	C16'—C15'—C14	114.7 (19)
C5—C4'—H4'2	110.1	C16'—C15'—H15C	108.6
C3'—C4'—H4'2	110.1	C14—C15'—H15C	108.6
H4'1—C4'—H4'2	108.4	C16'—C15'—H15D	108.6
C2—C3'—C4'	104.1 (12)	C14—C15'—H15D	108.6
C2—C3'—H3'1	110.9	H15C—C15'—H15D	107.6
C4'—C3'—H3'1	110.9	C15'—C16'—H16D	109.5
C2—C3'—H3'2	110.9	C15'—C16'—H16E	109.5
C4'—C3'—H3'2	110.9	H16D—C16'—H16E	109.5
H3'1—C3'—H3'2	109.0	C15'—C16'—H16F	109.5
C6—C5—C4	111.3 (4)	H16D—C16'—H16F	109.5
C6—C5—C4'	110.0 (8)	H16E—C16'—H16F	109.5
C6—C5—H5A	109.4	N2—C17—C18	111.4 (4)
C4—C5—H5A	109.4	N2—C17—H17A	109.3
C4'—C5—H5A	133.0	C18—C17—H17A	109.3
C6—C5—H5B	109.4	N2—C17—H17B	109.3
C4—C5—H5B	109.4	C18—C17—H17B	109.3
H5A—C5—H5B	108.0	H17A—C17—H17B	108.0
C6—C5—H5C	109.9	C19—C18—C17	113.1 (4)
C4'—C5—H5C	110.8	C19—C18—H18A	108.9
H5B—C5—H5C	130.8	C17—C18—H18A	108.9
C6—C5—H5D	108.9	C19—C18—H18B	108.9
C4—C5—H5D	131.5	C17—C18—H18B	109.0
C4'—C5—H5D	109.0	H18A—C18—H18B	107.8
H5C—C5—H5D	108.1	C18—C19—C20	112.1 (4)
C1—C6—C7	111.5 (3)	C18—C19—H19A	109.2
C1—C6—C5	120.8 (3)	C20—C19—H19A	109.2
C7—C6—C5	127.6 (3)	C18—C19—H19B	109.2
C8—C7—C6	112.3 (3)	C20—C19—H19B	109.2
C8—C7—C9	120.4 (3)	H19A—C19—H19B	107.9
C6—C7—C9	127.2 (3)	C19—C20—H20A	109.5
N1—C8—C7	122.5 (3)	C19—C20—H20B	109.5
N1—C8—S1	125.5 (2)	H20A—C20—H20B	109.5
C7—C8—S1	112.0 (2)	C19—C20—H20C	109.5
O2—C9—O1	121.9 (3)	H20A—C20—H20C	109.5
O2—C9—C7	124.6 (3)	H20B—C20—H20C	109.5
O1—C9—C7	113.5 (3)	C12—N1—C8	130.4 (3)
C11—C10—O1	111.0 (4)	C12—N1—H1A	121 (3)
C11—C10—H10A	109.4	C8—N1—H1A	109 (3)
O1—C10—H10A	109.4	C12—N2—C17	123.9 (3)
C11—C10—H10B	109.4	C12—N2—C13	120.2 (4)
O1—C10—H10B	109.4	C17—N2—C13	114.9 (3)
H10A—C10—H10B	108.0	C9—O1—C10	117.8 (3)
C10—C11—H11A	109.5	C8—S1—C1	90.86 (16)
			
C6—C1—C2—C3'	23.4 (10)	C6—C7—C9—O2	−179.4 (4)
S1—C1—C2—C3'	−155.7 (8)	C8—C7—C9—O1	−178.1 (3)
C6—C1—C2—C3	−9.1 (7)	C6—C7—C9—O1	0.8 (5)
S1—C1—C2—C3	171.9 (5)	N2—C13—C14—C15'	−145.7 (11)
C3'—C2—C3—C4	−57.1 (15)	N2—C13—C14—C15	171.8 (5)
C1—C2—C3—C4	39.8 (10)	C13—C14—C15—C16	76.9 (14)
C2—C3—C4—C5	−59.9 (11)	C15'—C14—C15—C16	−32.1 (19)
C1—C2—C3'—C4'	−54.3 (15)	C13—C14—C15'—C16'	−106 (3)
C3—C2—C3'—C4'	40.2 (10)	C15—C14—C15'—C16'	−19 (2)
C5—C4'—C3'—C2	71.5 (17)	N2—C17—C18—C19	177.6 (4)
C3—C4—C5—C6	45.6 (9)	C17—C18—C19—C20	172.7 (4)
C3—C4—C5—C4'	−48.1 (16)	N2—C12—N1—C8	171.8 (3)
C3'—C4'—C5—C6	−50.6 (14)	S2—C12—N1—C8	−7.6 (5)
C3'—C4'—C5—C4	47.6 (13)	C7—C8—N1—C12	−172.7 (3)
C2—C1—C6—C7	−179.1 (4)	S1—C8—N1—C12	5.7 (5)
S1—C1—C6—C7	0.0 (4)	N1—C12—N2—C17	−7.3 (5)
C2—C1—C6—C5	−1.7 (6)	S2—C12—N2—C17	172.2 (3)
S1—C1—C6—C5	177.4 (3)	N1—C12—N2—C13	−175.5 (3)
C4—C5—C6—C1	−15.9 (6)	S2—C12—N2—C13	3.9 (5)
C4'—C5—C6—C1	17.0 (8)	C18—C17—N2—C12	−81.1 (5)
C4—C5—C6—C7	161.0 (5)	C18—C17—N2—C13	87.7 (4)
C4'—C5—C6—C7	−166.1 (7)	C14—C13—N2—C12	−92.5 (5)
C1—C6—C7—C8	0.8 (4)	C14—C13—N2—C17	98.3 (5)
C5—C6—C7—C8	−176.3 (3)	O2—C9—O1—C10	−0.9 (6)
C1—C6—C7—C9	−178.1 (3)	C7—C9—O1—C10	178.9 (3)
C5—C6—C7—C9	4.8 (6)	C11—C10—O1—C9	−92.2 (5)
C6—C7—C8—N1	177.3 (3)	N1—C8—S1—C1	−177.4 (3)
C9—C7—C8—N1	−3.7 (5)	C7—C8—S1—C1	1.1 (3)
C6—C7—C8—S1	−1.3 (4)	C6—C1—S1—C8	−0.7 (3)
C9—C7—C8—S1	177.7 (3)	C2—C1—S1—C8	178.5 (3)
C8—C7—C9—O2	1.8 (6)		

### Hydrogen-bond geometry (Å, °)

**Table:**

*D*—H···*A*	*D*—H	H···*A*	*D*···*A*	*D*—H···*A*
N1—H1A···O2	0.86 (3)	1.89 (2)	2.643 (4)	145 (3)
